# Protocol for the Addressing the Social Determinants and Consequences of Tuberculosis in Nepal (ASCOT) pilot trial

**DOI:** 10.12688/wellcomeopenres.17669.3

**Published:** 2022-12-13

**Authors:** Bhola Rai, Kritika Dixit, Raghu Dhital, Poonam Rishal, Suman Chandra Gurung, Puskar Raj Paudel, Gokul Mishra, Laura Bonnett, Noemia Siqueira-Filha, Mukti Nath Khanal, Knut Lonnroth, S Bertel Squire, Maxine Caws, Tom Wingfield

**Affiliations:** 1 Research, Birat Nepal Medical Trust, Kathmandu, 44600, Nepal; 2 WHO Collaborating Centre on Tuberculosis and Social Medicine, Karolinska Institute, Stockholm, 171 77, Sweden; 3 Planning, Monitoring, Evaluation, Surveillance, and Research, Nepal Tuberculosis Control Centre, Kathmandu, 44600, Nepal; 4 Institute of Population Health, University of Liverpool, Liverpool, L69 3GF, UK; 5 Health Sciences, University of York, UK, York, YO10 5DD, UK; 6 Clinical Sciences, Liverpool School of Tropical Medicine, Liverpool, L3 5QA, UK; 7 Clinical Sciences and International Public Health, Liverpool School of Tropical Medicine, Liverpool, L3 5QA, UK; 8 Tropical and Infectious Diseases Unit, Liverpool University Hospital NHS Foundation Trust, Liverpool, UK

**Keywords:** Tuberculosis; poverty; social determinants; catastrophic costs; stigma; social protection; socioeconomic support; End TB Strategy; pilot trial; process evaluation; mixed-methods.

## Abstract

**BACKGROUND: **The World Health Organization’s End TB (tuberculosis) Strategy advocates social and economic support for TB-affected households but evidence from low-income settings is scarce. We will evaluate the feasibility and acceptability of a locally-appropriate socioeconomic support intervention for TB-affected households in Nepal.

**METHODS: **We will conduct a pilot randomised-controlled trial with mixed-methods process evaluation in four TB-endemic, impoverished districts of Nepal: Pyuthan, Chitwan, Mahottari, and Morang. We will recruit 128 people with TB notified to the Nepal National TB Program (NTP) and 40 multisectoral stakeholders including NTP staff, civil-society members, policy-makers, and ASCOT (Addressing the Social Determinants and Consequences of Tuberculosis) team members. People with TB will be randomised 1:1:1:1 to four study arms (n=32 each): control; social support; economic support; and combined social and economic (socioeconomic) support. Social support will be TB education and peer-led mutual-support TB Clubs providing TB education and stigma-reduction counselling. Economic support will be monthly unconditional cash transfers during TB treatment with expectations (not conditions) of meeting NTP goals. At 0, 2, and 6 months following TB treatment initiation, participants will be asked to complete a survey detailing the social determinants and consequences of TB and their feedback on ASCOT. Complementary process evaluation will use focus group discussions (FGD), key informant interviews (KII), and a workshop with multi-sectoral stakeholders to consider the challenges to ASCOT’s implementation and scale-up. A sample of ~100 people with TB is recommended to estimate TB-related costs. Information power is estimated to be reached with approximately 25 FGD and 15 KII participants.

**CONCLUSIONS:** The ASCOT pilot trial will both generate robust evidence on a locally-appropriate, socioeconomic support intervention for TB-affected households in Nepal and inform a large-scale future ASCOT trial, which will evaluate the intervention’s impact on catastrophic costs mitigation and TB outcomes.

The trial is registered with the ISRCTN ( ISRCTN17025974).

## Introduction

Tuberculosis (TB) kills 4100 people per day worldwide. In 2020, approximately 10 million people developed TB disease, nearly 3 million of whom were not notified to national TB programmes or were not diagnosed and treated ^1^. Stigma, marginalization, catastrophic costs of accessing TB healthcare services, and lack of social protection, can lead to diagnostic delay, worsen TB treatment outcomes, and compound poverty amongst TB-affected households, especially in low- and middle-income countries (LMICs) ^2, 3^.

To progress towards ending TB, within the framework of the United Nations Sustainable Development Goals (SDGs), the World Health Organization’s (WHO) 2015 End TB Strategy set mandated that “Zero TB-affected families should face catastrophic costs” (total TB-related costs >20% of annual household income) and that TB-affected people should be provided with psychosocial and economic (socioeconomic) support ^4^. However, there is limited evidence from LMICs on the optimal strategies to deliver socioeconomic support to TB-affected households.

In Peru, members of our project team identified a catastrophic costs threshold, now used as WHO’s global catastrophic costs indicator, above which TB patients were more likely to abandon treatment or die ^5^; successfully trialled a novel socioeconomic intervention that mitigated catastrophic costs, improved uptake of TB preventive therapy and increased rates of cure in TB-affected households ^6– 8^; and contributed to generation of risk scores that accurately identified TB contacts and households at greatest risk of being affected by TB disease ^9, 10^.

However, Peru is classified as a middle-income country and has well established cash-transfer schemes, which limits generalizability of these encouraging findings. Therefore, it is vital to develop, implement, and evaluate interventions similar to those in Peru in other settings, especially TB-endemic LMICs with limited social protection coverage. We aimed to address this research gap in Nepal, a LIC with substantial rates of TB and poverty.

In 2018, we won a Wellcome Seed Award (grant number 209075/Z/17/Z) to conduct preliminary research to design a socioeconomic support intervention suitable for TB-affected households in Nepal ^11^. The Seed Award research used mixed methods to characterise the socioeconomic impact and barriers to accessing TB diagnosis and care in Nepal and to support design of potential interventions to overcome these issues ^12^. The Seed Award was housed within the “ IMPACT-TB” active-case finding study in Vietnam and Nepal (EU-Horizon 2020, grant 733174).

**Figure 1.**
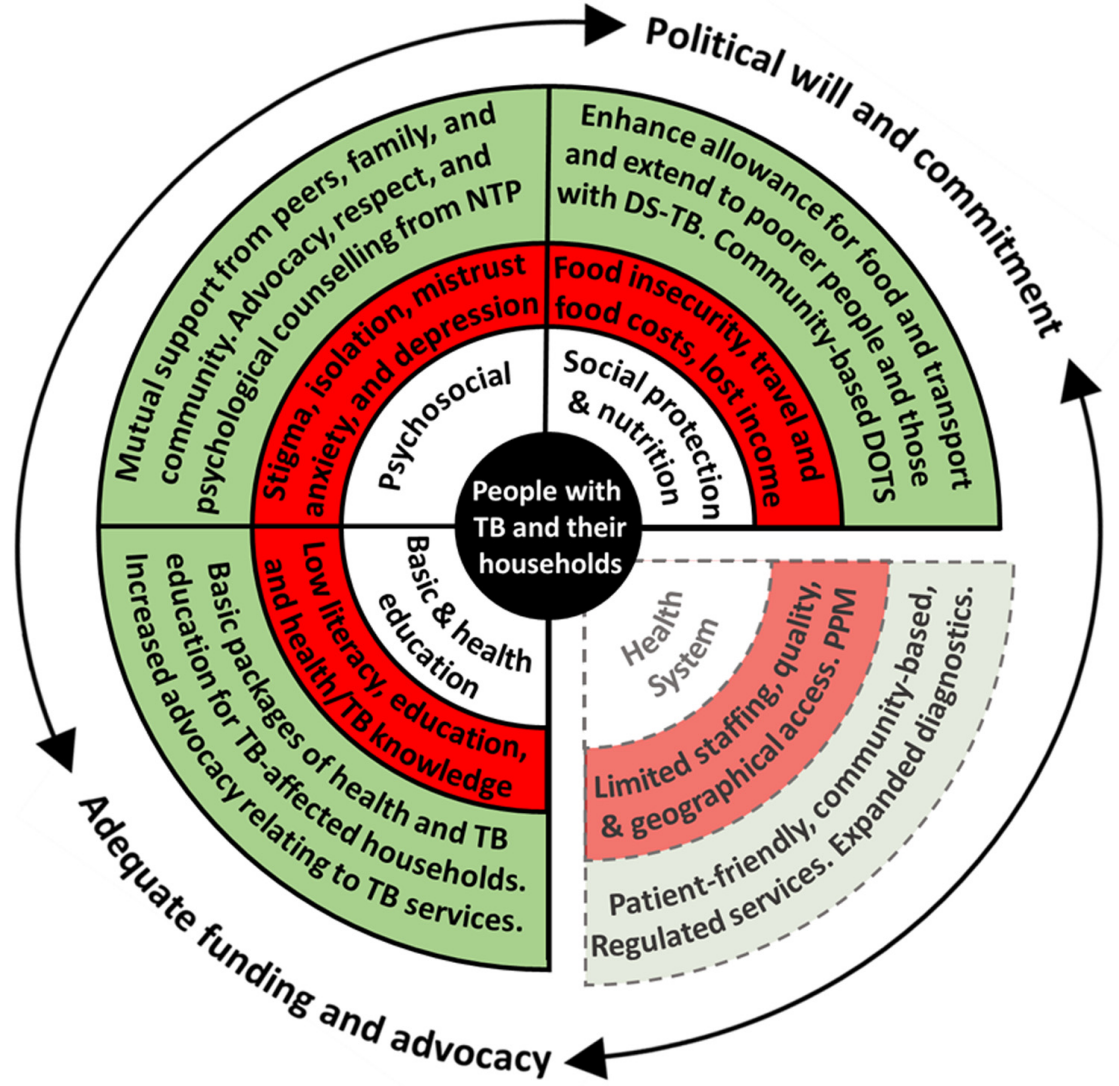
Perceived barriers and facilitators to accessing tuberculosis (TB) services in Nepal. Legend: The inner white circle contains the key categories that influence TB service access and engagement, which are adapted from a World Health Organization Medication Adherence Framework ^17^. The middle red circle indicates the main barriers identified for each category, which may threaten access to TB services. The outer green circle indicates the main facilitators (current or potential) for each category, which may enhance access to TB services. Barriers relating to “TB, health, and basic education”, “Social protection and nutrition”, and “Psychosocial” were perceived by the project team to be modifiable by a household level socioeconomic intervention. Barriers relating to the “Health System” were perceived by the project team to be non-modifiable by a household level socioeconomic intervention and are, therefore, separated from the other categories and represented by dotted lines. “PPM” is “Public Private Mix” and, as a health system barrier, refers to the protracted and convoluted patient journey through public and private healthcare providers, which was reported as being associated with increased economic impact, especially related to out-of-pocket costs. The surrounding arrows indicate the cross-FGD finding that adequate funding and advocacy, and political will and commitment were perceived as vital structural factors to enable the facilitators identified to overcome the barriers identified. Note: This figure and legend are reproduced with permission from a paper by Dixit *et al.* published in BMJ Open under a CC BY public copyright license ^18^. Abbreviations: DS-TB = drug-sensitive TB; NTP = National TB Program; and TB = tuberculosis.

**Figure 2.**
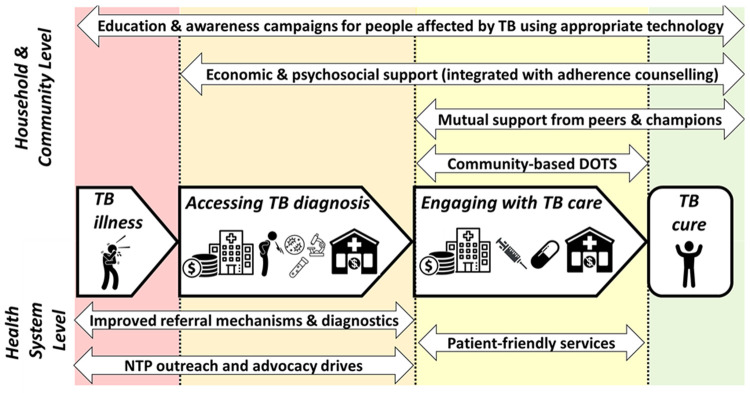
Facilitators along the tuberculosis (TB) patient pathway. Legend: The figure shows the facilitators identified to support people with TB throughout their patient journey (central arrows in bold) to get diagnosed promptly, engage with care, and become cured, and their households to avoid catastrophic costs. The upper section shows facilitators that are achieved at household and community level and so could potentially be included in design of a household-level support intervention for TB-affected households for trial evaluation. The lower section shows facilitators that are achieved at the health system level and would not be included in design of a TB-affected household-level support intervention. The two headed arrows in upper and lower section show the specific facilitators identified and the length of the arrow indicates which stages along the patient pathway the intervention could influence or modify. Figure and legend reproduced with permission from Dixit *et al.* ^14 ^poster presentation PS-33-C8 at the 50 ^th^ Union World Conference on Lung Health International Union Against Tuberculosis and Lung Diseases Annual General Meeting in Hyderabad, India. Abbreviations: NTP = National TB Program; TB = tuberculosis.

Both the Seed Award and IMPACT-TB were implemented by a well-established Nepalese NGO, Birat Nepal Medical Trust ( BNMT), with over 50 years of experience in implementing public health interventions in Nepal. IMPACT-TB and the Wellcome Seed Award research found that catastrophic costs were experienced by 61% of TB-affected households ^13^ and identified distinct socioeconomic factors impeding access to TB services ( Figure 1) ^14^. Focus group discussions (FGDs) characterised where along the patient pathway from TB symptoms to care seeking, diagnosis, treatment, and treatment outcome, support interventions could be delivered ( Figure 2). The research also demonstrated that people with TB in Nepal commonly experienced self-stigma ^15^ and exploratory analyses suggested that experiencing stigma was associated with catastrophic costs and worse TB treatment outcomes ^16^.

The culmination of the Wellcome Seed Award research was a multisectoral participatory workshop. During the workshop, participants created and voted on a list of socioeconomic support interventions for households affected by TB in Nepal that were perceived as suitable for further trial evaluation ^11^. Participants opted for an intervention of TB education and stigma counselling during a household visit, and unconditional monthly cash transfers (see Rai *et al.* ^11^ for more detail).

## Protocol

### Ethical statement

The research ethics committees of the Liverpool School of Tropical Medicine, UK, (approval number 20–098) and the National Health Research Council of Nepal (NHRC) research ethics committee (approval number 363/2021) approved the study in September 2021. NHRC will make at least one site visit and audit of ASCOT project documents during the lifetime of the ASCOT pilot trial.

All study participants will be provided with participant information leaflets prior to completion of written informed consent (see Extended data ^19^).

Informed consent will adhere to Good Clinical Practice (GCP) principles and relevant regulatory requirements nationally and internationally. It will be initiated before an individual agrees to participate in the ASCOT pilot trial and will continue throughout that individual’s participation. Discussion of the conditions, objectives, risks and inconveniences of this research will be provided to the participant by ASCOT team members with training in informed consent processes and procedures including the Participant Information Sheet (PIS). The PIS reiterates that trial participation is voluntary and withdrawal from the trial is possible at any time and for any reason. Time will be given by the team member taking consent to ensure that the participant or delegate for the participant has ample opportunity to consider the information, pose questions and discuss the ASCOT pilot trial with friends, family members, and/or others as required.

The team will liaise closely with the relevant local NTP staff throughout the project. To examine treatment outcomes during the trial, participants’ NTP medical records will be photocopied from the Nepal NTP register. The photocopies will obscure the participant’s identifiable details and a unique participant study number identifier added.

### Trial registration

The ASCOT Pilot Trial is registered with the International Standard Randomised Controlled Trial Number website ( ISRCTN17025974) ^20^ and the protocol adheres to – and subsequent project reports will adhere to - the SPIRIT 2013 Statement where applicable ^21^.

### Study design

ASCOT is a randomised-controlled pilot trial and mixed-methods process evaluation of a socioeconomic intervention for people with TB and their households in Nepal. The pilot trial is designed to allow adaptation of the intervention in real time during its implementation. Adaptive randomised-controlled design is recommended by the Medical Research Council, UK, and is the optimal method to generate new knowledge on the implementation and impact of the socioeconomic support intervention. This mixed-methods process evaluation of a pilot trial will: provide rich data; better understand context, causal mechanisms, and generalisability; reduce Type III errors (dismissing a potentially impactful intervention due to implementation failure); and improve design and delivery of the definitive, future trial ^22^.

### Co-primary aims

The co-primary aims of the ASCOT pilot trial are to evaluate:

- the feasibility of a socioeconomic support intervention for TB-affected households in Nepal from a provider perspective; and
- the acceptability of a socioeconomic support intervention for TB-affected households in Nepal including from both a client perspective and a health system readiness and scalability perspective.

### Secondary aims

Secondary aims will be exploratory and include:

- assessment of the impact of the intervention on TB treatment outcomes, stigma, mental health, and catastrophic costs;
- generation of an open-access project manual detailing the preparatory mixed-methods research with key stakeholders, pilot trial methods, and implementation costs; and
- informing design and increase likelihood of funding and implementation success of a large-scale randomised controlled trial of socioeconomic support for TB-affected households in Nepal.

Exploratory aims are to:

- assess the impact of the intervention on TB treatment outcomes, stigma, mental health, and catastrophic costs;
- generate an open-access project manual detailing the preparatory mixed-methods research with key stakeholders, pilot trial methods, and implementation costs; and
- inform design and increase likelihood of funding and implementation success of a large-scale randomised controlled trial of socioeconomic support for TB-affected households in Nepal.

**Figure 3.**
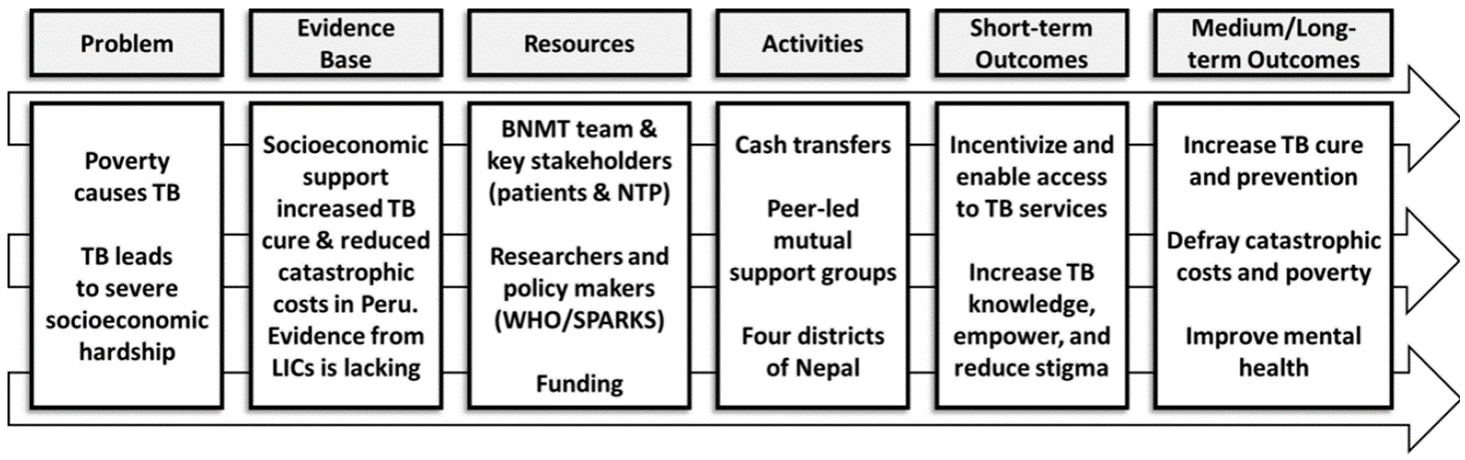
ASCOT Logic Model. Abbreviations: ASCOT = the “Addressing the Social Determinants and Consequences of Tuberculosis” project; BNMT = Birat Nepal Medical Trust; LICs = Low-income countries; SPARKS = Health and Social Protection Action Research Knowledge Sharing network; TB = tuberculosis; and WHO = World Health Organization.

The planned larger, well-powered, follow-on randomised controlled trial will have short-term outcomes including increased access to TB services and longer-term outcomes including increased TB treatment success, mitigation of catastrophic costs, reduced self-stigma, and improved mental wellbeing among people with TB (see ASCOT Logic Model, Figure 3).

### Study setting

Nepal is a lower middle-income country with a TB incidence of 245/100,000 people per year ^23^. Moreover, TB is the seventh leading cause of death in Nepal ^24^. Multi-drug resistance (MDR) rates are low in new cases but affect approximately 1 in 7 people being retreated for TB. Rates of treatment success are over 90% but lower for people with MDR-TB (70%) and very low for people with HIV-TB co-infection (9%). Treatment coverage is 70% indicating difficulties with access to TB services. This is compounded by stigma and discrimination towards people with TB and self-stigmatisation, which have been found to be prevalent in cohort studies in Nepal ^14, 15, 25^.

The National Tuberculosis Program (NTP) strategy focuses on a biomedical approach to address TB, including recent advances in expanded access to molecular diagnostic tests such as Xpert MTB/RIF ^®^^24^. Nevertheless, despite free basic TB diagnostic tests and medicines and financial support for people with MDR-TB, more than half of TB-affected people experience catastrophic costs (defined as total TB-related costs and lost income >20% of a household’s annual income) of TB illness and care seeking in Nepal. Such costs include travel for Directly Observed Therapy (DOT), expenses for additional nutrition, and loss of income, and can lead to lower rates of TB treatment success, especially amongst underserved households ^13, 25– 27^. To address this, Objective 1 of the Ministry of Health and Population National TB Control Centre’s National Strategic Plan to End Tuberculosis in Nepal, 2021–2026 is “to strengthen the health system and improve quality TB services under universal health coverage and ensure no TB-affected family faces catastrophic costs due to TB by 2025”.

Fewer than one in two people in Nepal are covered by a basic social protection floor and one in four live in poverty ^28^. Despite widespread use of DOT, greater GeneXpert coverage, and intensified case finding, TB services in Nepal are challenged by significant geographical barriers, including mountains, flooding, landslides, and poor road infrastructure, all of which can hamper TB prevention and treatment outcomes ^24, 26– 29^. The Nepal NTP provides 3,000 Nepalese Rupees (~27 USD) monthly cash transfers to people with MDR-TB receiving ambulatory care, nominally for nutrition and transport ^24^. People with drug-sensitive TB (DS-TB) do not currently receive any financial support in Nepal ^11^.

**Figure 4.**
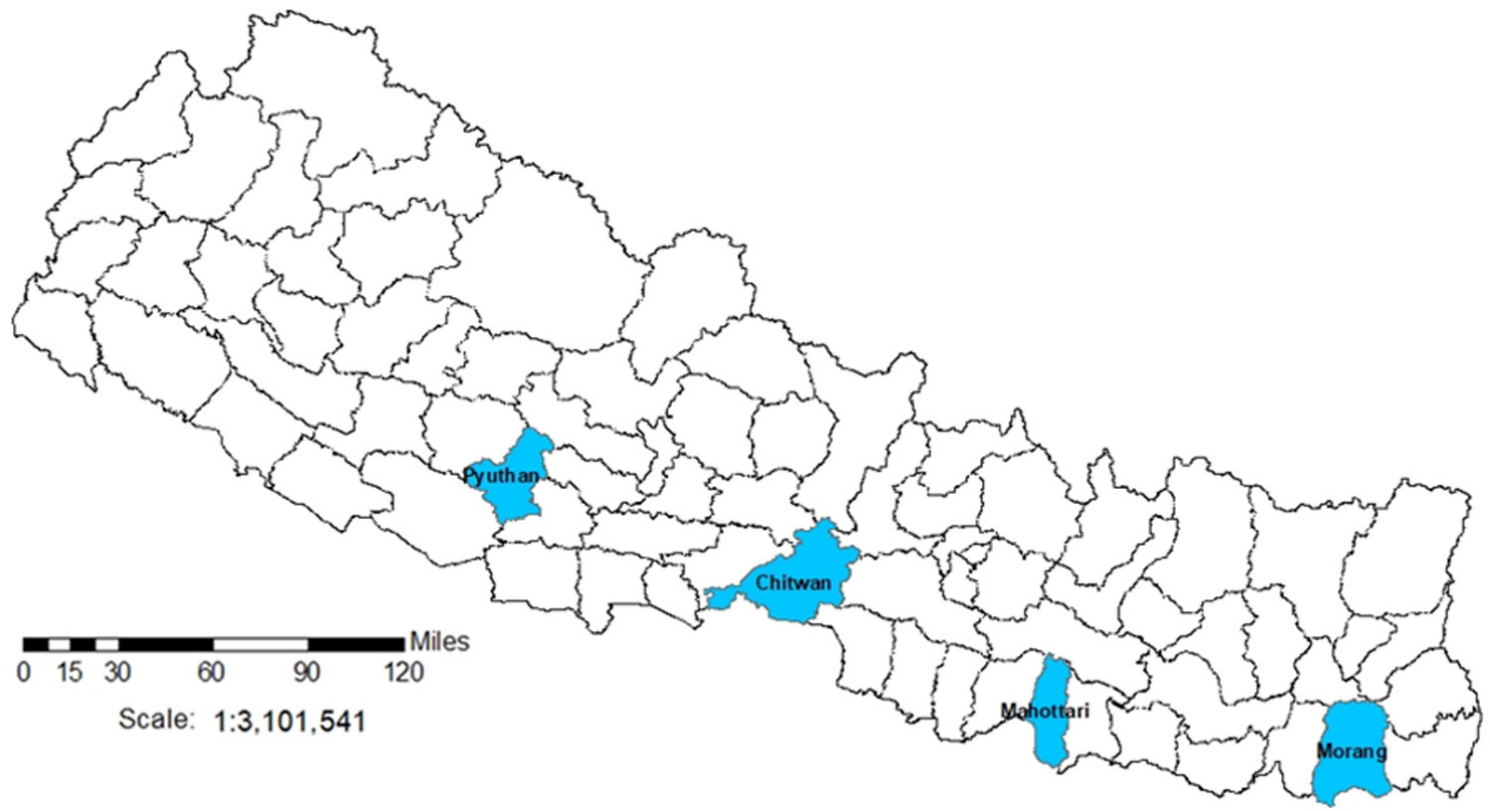
ASCOT Study Sites in Nepal. Legend: The figure shows the 77 second-level administrative country subdivision districts of Nepal. Blue shading indicates the ASCOT study sites.

The ASCOT Pilot Trial will take place in four districts of Nepal purposively selected due to similar TB and poverty profiles: Pyuthan, Chitwan, Mahottari, and Morang ( Figure 4). In these districts, over 35% of people live below the poverty line, 40% are undernourished, and TB incidence is ~150/100,000 people ^30^. Moreover, the 2019 national prevalence survey showed more than half of TB cases never reach TB services, including due to social and economic barriers to access.

### Study population

***Participant identification, recruitment, and follow-up***

There will be two study populations involved in ASCOT. The first will be the people with TB diagnosed by active- (ACF) and passive case-finding (PCF), recruited to the pilot trial and process evaluation, who will be randomised to an ASCOT study arm following recruitment. The second will be multisectoral, key stakeholders recruited to participate in the complementary enhanced process evaluation, which will evaluate not only acceptability and feasibility but also health systems readiness. The two populations are detailed separately below.

***Pilot trial participant population***

Each of the four study sites will consecutively recruit 32 people with TB to reach the required pilot trial sample size of 128 people. Potential participants will be invited to participate during attendance at routine NTP TB services and clinics with a specific informed consent form and participant information sheet (see Extended data ^19^).

Inclusion criteria for the pilot trial participants are: a person aged 18 years or older with microbiologically-confirmed TB notified to the Nepal National TB Program (NTP) and registered in the TB register of a TB clinic within the study site district, whether diagnosed through ACF or PCF; and able to provide written, informed consent or assent (or thumb print if unable to write). The project team will be aware of the participant’s TB resistance profile but the profile itself does not form a part of the eligibility criteria to participate unless a participant has confirmed multi-drug resistant TB (MDR-TB), which is associated with a distinct treatment pathway including admission to an MDR-TB hostel in the initiation phase of treatment. In the study sites, resistance to rifampicin and/or isoniazid will usually be reported within 24–48 hours of initial TB diagnosis using GeneXpert molecular testing and then confirmed by phenotypic sensitivity testing.

Exclusion criteria for the pilot trial participants are: a person aged under 18 years of age; another member of the potential trial participant’s household is already a participant in the ASCOT study; a person not notified to Nepal National TB Program and not registered in the TB register of a TB clinic within the study site district; and a person who is unable to provide written, informed consent or thumb print. In addition to the above, pilot trial participants who receive an alternative diagnosis during their treatment (e.g. diagnosis of TB rescinded and, in some cases, alternative non-TB diagnosis made) and are removed from the NTP register will also be excluded from further follow-up.

***Enhanced process evaluation participant population***

During our previous Wellcome Seed Award research, we performed a desk-based scoping exercise to identify key in-country stakeholders in Nepal with expertise in and/or experience of TB and social protection, which is described in further detail in Dixit *et al.* ^12^ We will update this scoping exercise to ensure a current and relevant list of potential stakeholders is produced. The stakeholders will be: civil-society organisation (CSO) representatives including from cooperatives, women’s groups, and grass roots organisations; community leaders (e.g. district elders); social-protection decision makers; NTP leaders and managers; and NTP multi-disciplinary staff, predominantly from the study sites. Additional stakeholders will be ASCOT field team members including community health supervisors (CHSs), female community health volunteers (FCHVs), and District Program Coordinators (DPCs); and a subset of people with TB recruited to each arm of the ASCOT study will be purposively selected. Purposive selection will aim to achieve representation by gender, age, poverty level, comorbidities (e.g. HIV), and in the case of participants, ASCOT arm. 

The following stakeholders will be invited to participate in separate focus group discussions (FGDs):

- Civil Society Organisation (CSO) representatives (n=5)
- Community leaders (n=5)
- NTP multi-disciplinary staff (n=5)
- ASCOT Community Health Supervisors (CHS) (n=5)
- ASCOT Female Community Health Volunteers (FCHVs) (n=5)
- People with TB (separate FGDs of n=5 with participants from each study arm, including those found by ACF and PCF)

The following stakeholders will be invited to participate in key informant interviews (KIIs):

- NTP managers (n=5)
- Social protection decision makers (n=5)
- ASCOT District Program Coordinators (DPCs) (n=5)

Inclusion criteria for the process evaluation participants are: aged 18 years or older; identified by scoping review or, if a person with TB participated in ASCOT pilot trial whether recruited by ACF or PCF; able to provide written, informed consent (or thumb print if unable to write).

Exclusion criteria for the process evaluation participants are: under 18 years of age; not identified by scoping review or, if a person with TB did not participate in ASCOT pilot trial; and unable to provide written, informed consent or thumb print.

***Sample size and statistical power***

A sample of ≥40 people is recommended for pilot trials and process evaluations ^31^ and ~100 people for TB patient costs data ^32^. Our previous research in the study site attained recruitment rates of >90% of those invited, attrition during follow-up was less than 5%, and we anticipate similar rates during the ASCOT project ^15^. We have budget allowance for minor coronavirus disease 2019 (Covid-19) under-recruitment or attrition and plan to continue recruitment of people with TB until we recruit 128 participants to ensure the sample size of 100 with complete data available is met.

Information power (saturation) is estimated to be reached at 25 FGD and 15 KII participants ^33^. Purposive selection of participants and implementers for FGDs and KIIs will ensure gender, district, poverty, and NTP/project role diversity. Previous uptake of FGDs and KIIs was good but we will invite approximately 50 stakeholders as contingency.

### Study activities and interventions

ASCOT study activities can be broadly divided into: pilot trial activities and interventions with recruited people with TB and their households, which will gather data to support process evaluation; and complementary enhanced process evaluation activities with a subset of people with TB and key multisectoral stakeholders. 

***Pilot trial***

*Recruitment of people with TB and their households*

The ASCOT team will support BNMT DPCs, CHS, and FCHVs to recruit 128 people with TB across study sites. Participants will be identified prospectively during the recruitment period once they are diagnosed and notified to the NTP register. Participants will be invited to participate at their initial TB clinic visit, usually prior to initiation of treatment. They will then be randomised to a study arm at that clinic visit.

Household contacts of participants in social and socioeconomic study arms will be invited to participate in the social support activities using index-patient initiated invitations, which has been reported to be a preferred recruitment method for household studies in multiple settings ^34– 36^. Specifically, people with TB recruited to the study and randomised to the social or socioeconomic arms will be given an information leaflet and advised to inform their household members about the social support activities. As per WHO guidance, a household contact will be defined as “a person who shared the same enclosed living space for one or more nights or spent frequent or extended periods during the day with the index case during the 3 months prior to commencement of the current treatment episode” ^37^. Household contacts will also be given a separate PIS and asked to complete separate consent forms.

Participants will be free to withdraw from the study at any time by indicating this desire verbally and/or in writing to a member of the project team. Individual-level data from participants who withdraw will not be used further and will be securely deleted from project records. Deidentified group-level data collected during activities such as Focus Group Discussions, in which a participant who later withdraws from the study took part, will remain available for analysis.

*Household visits and surveys*

All participants will receive a household visit at three time points to complete the ASCOT survey:

- **Visit one:** two to four weeks after TB treatment initiation, during the “intensive treatment phase”;
- **Visit two:** eight to 12 weeks after TB treatment initiation, during the “continuation treatment phase”; and
- **Visit three:** 20 to 24 weeks following TB treatment initiation, to coincide with the completion of a standard 6-month course of DS-TB treatment.

This is line with WHO methods, which require a person with TB to have been taking treatment in a specific phase for at least two weeks before answering questions relating to the socioeconomic impact of that phase ^38^. During household visits, a survey adapted from WHO’s TB Patient Costs Survey ^5, 38^ and previously used in more than 400 people with TB in Nepal ^13, 16, 39^, will be used to interview the participant with TB and, where appropriate, their family members. During the Wellcome Trust Seed Award research, the survey was translated from English into Nepali before back translation into English to check consistency. It was then evaluated by the ASCOT study team and piloted in Nepali language with 20 people with TB in the study sites, refined, and validated for field deployment.

The survey will collect data on:

i) Poverty status using characteristics including assets, housing, and amenities ^5, 6, 33^;
ii) Food insecurity and hunger;
iii) Direct and indirect costs of accessing and engaging with TB care (see Rai *et al.*) ^12^;
iv) coping strategies including dissaving (e.g. selling assets), schooldays lost, and temporary income-generating activities;
v) TB-related knowledge adapted from a WHO TB Knowledge Attitudes and Practices survey, and including knowledge of transmission, prevention, and treatment of TB;
vi) Mental health including depression measured by an adapted PHQ9 previously validated in Nepal, a resilience scale, and the EuroHIS-QoL ^12, 17, 40^;
vii) Psychosocial situation evaluated through questions relating to social capital and self-reported perceived stigma measured using an adapted version of the Stop TB Partnership stigma assessment tool ^41, 42^; and
viii) Their feedback on the ASCOT project and support they had received, evaluated through use of both closed ranking user-satisfaction Likert Scale and open questions with qualitative, free text responses, adapted from our previous research ^43^.

The data collected above will add value to the study as it can be integrated with data from 400 previously recruited patients for spin-off analyses. In addition to the above, with permission of both NTP and participants, data on TB treatment outcomes will be collated from NTP registry data. This will include WHO-defined definitions of TB treatment outcome including treatment success, treatment failure, loss to follow up, and death. This data will support exploratory analysis of the effect of socioeconomic support on TB treatment outcomes.

**Figure 5.**
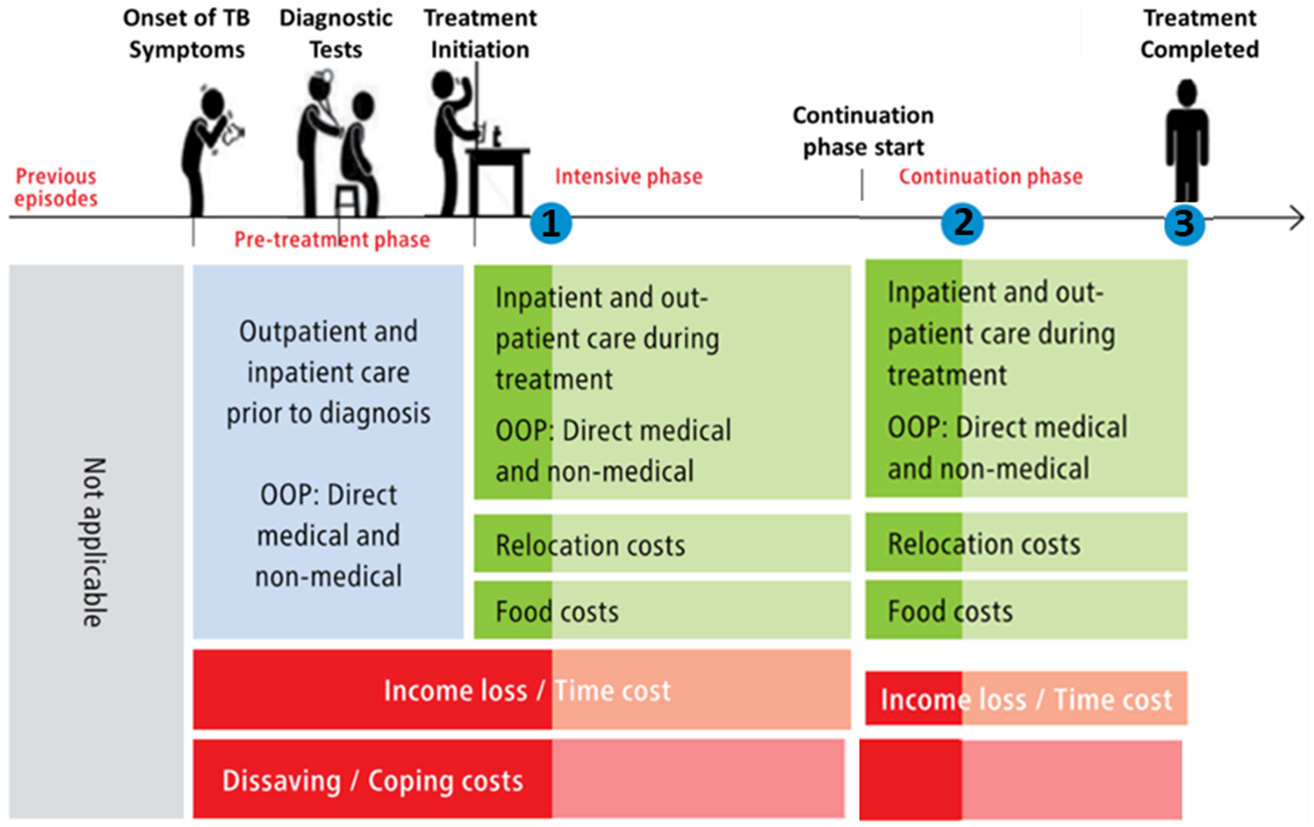
Household visit and survey timing, and economic data collected ^38^. Legend: The blue dot indicates the timing of household visits one (1), two (2), and three (3). Lighter shades of green and red indicate data not collected at visit 1 and 2 respectively. Abbreviations: OOP = out-of-pocket costs; and TB = tuberculosis.

Figure 5 summarises the economic information gathered during each visit. 

*Study arms and activities*

**Figure 6.**
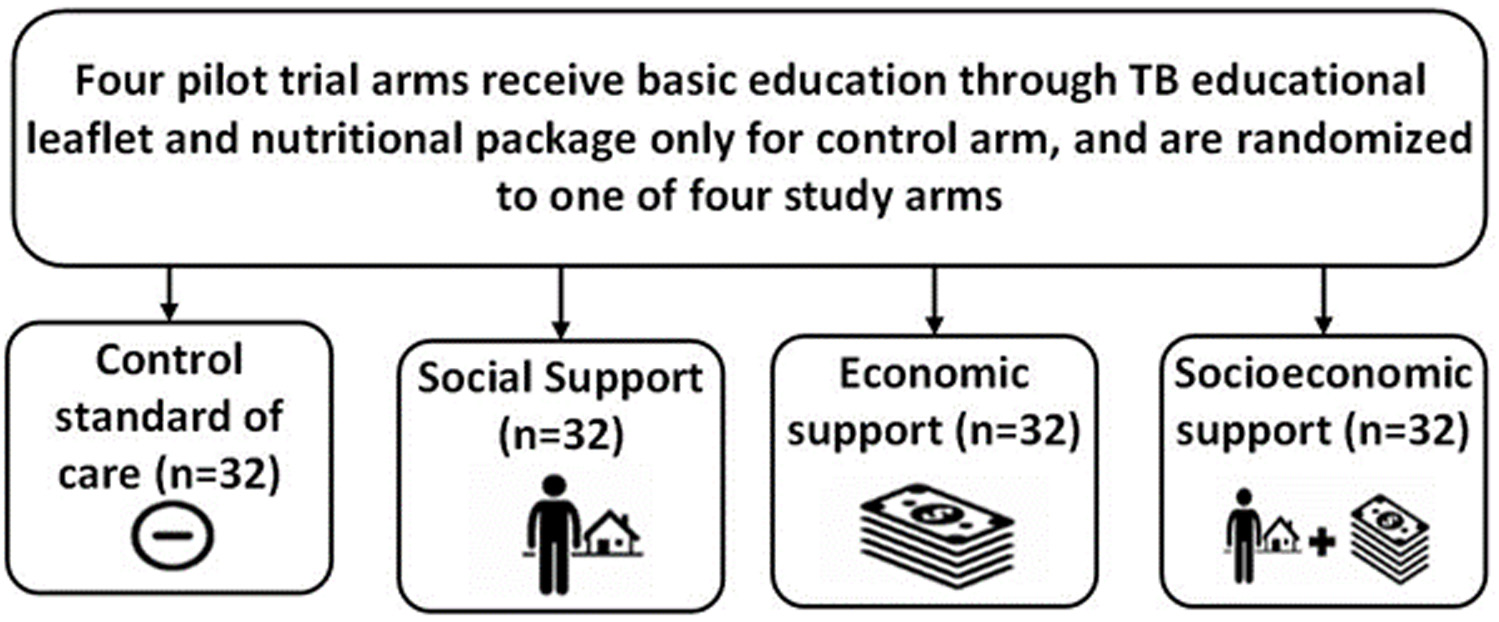
Study arm allocation (abbreviations: TB = tuberculosis).

All participants will complete the survey but receipt of study interventions will depend on which of the four study arms the participant and their household are randomly allocated to ( Figure 6).

In line with our previous research ^7^ and BNMT work, during an initial household visit, *all* participants and their households will receive a basic TB and health educational package delivered by trained BNMT CHS and/or FHCVs and/or a TB survivor where available and a one-off nutritionally-optimised nutritional packages with a value of approximately 3000 Nepalese rupees / 18 GBP. Contents of the packages were previously suggested at a related workshop with in-country stakeholders in Nepal in September 2019 ^11^.

**Figure 7.**
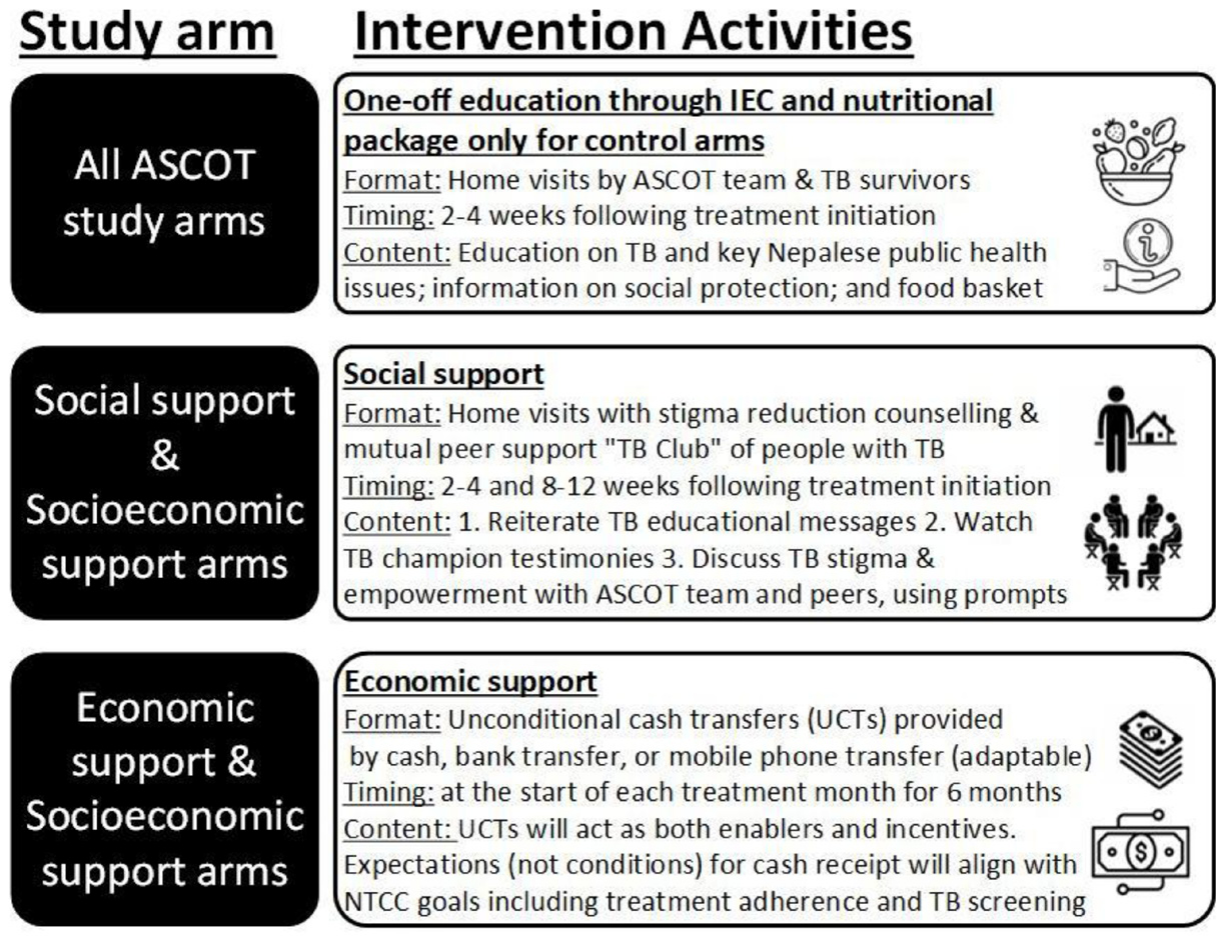
Intervention activities conducted in each study arm. Abbreviations: ASCOT = the “Addressing the Social Determinants and Consequences of Tuberculosis” project; IEC = Information, Education, and Counselling; NTCC = Nepal National Tuberculosis Control Centre; TB = tuberculosis; UCT = unconditional cash transfer.

Participants in support arms will receive either a social support intervention, economic support intervention, or integrated socioeconomic support intervention. Figure 7 gives further details of activities in each intervention. Interventions will be piloted in approximately 2–3 recruited TB-affected households per study site. ASCOT team members will review the implementation of intervention after this initial pilot and may adapt or refine activities within the intervention. This could include adaptations in dose (e.g. frequency of economic or social support) and mode or mechanism of delivery (e.g. mechanism of economic support could be phone, cash, or bank transfer; social support could be delivered at home, in a community setting, or both). At this “decision point” juncture, the interim progress review findings will be shared with the TSC and a TSC meeting scheduled to discuss these findings. Expectations for cash transfer receipt will be aligned with Nepal NTP strategy goals including regular adherence to TB medication and household contacts attending TB clinics for screening.

Following refinements by BNMT team and TSC inputs, recruitment will proceed. Again, when a study site reaches 32 recruited participants, recruitment will discontinue in that district. This provides ample allowance for completion of intervention activities and three surveys in 32 participants given the predicted attrition rate of ≤10% in the study sites.

The longitudinal survey data detailing participant feedback will form part of the process evaluation of the acceptability and feasibility of the ASCOT intervention. This will be supplemented by real-time self-reported ASCOT project feedback, and time and expense diaries of BNMT implementers, including DPCs and CHS. This data will be further complemented by the enhanced process evaluation aiming to evaluate health systems readiness and scalability of the ASCOT intervention. 

There will be multiple opportunities during the implementation of the pilot trial to assess for any harms or adverse effects. First, the longitudinal survey includes questions related to feedback from participants on the interventions received and involvement in the ASCOT project. Second, after each TB Club is conducted, participants will be asked to provide verbal and written feedback on the activities undertaken during the TB Club, including whether they perceive any of the activities to have caused harm or have the potential to cause harm. Third, issues relating to harm or adverse effects of the ASCOT project are included in the topic guides for KIIs and FGDs. All of these data will be analysed in real-time during the project and discussed between the ASCOT project team and where necessary the TSC to identify if any remediating actions are required. The criteria for discontinuing interventions include: participants who withdraw from the ASCOT project who, as an individual and inclusive of their household, will no longer receive the intervention; harm or adverse effect is identified that is felt by the project team and TSC to preclude continuation of interventions; and acts of God or unforeseen cuts to the study budget curtailing project activities.

***Complementary enhanced process evaluation***

A process evaluation will use and develop our mixed methods research techniques ^2, 16, 33, 44^ including FGDs, KIIs, and a participatory workshop. The process evaluation will also be enhanced and complemented by an assessment of health systems readiness to deliver the ASCOT intervention and potential scalability of the intervention into routine NTP practice in Nepal.

*Health systems readiness and scalability*

Even effective interventions will *not* have impact unless they can be scaled-up and integrated into organizational practices such as care pathways, and normalised into health professionals’ routine practice ^45^. The process evaluation will include both a health systems readiness and scalability assessment. The concept of scalability is relatively new in implementation science ^46^. It is often thought to be interchangeable with the ability to widen the reach of an intervention. However, there are many more considerations and a lack of attention given to how the intervention will perform under routine conditions or the extent to which it is embedded in a local delivery system will hinder not only the implementation but also the possibility for scale-up. Therefore, it is of paramount importance to assess the potential to scale-up early in the process ^47^. Prior to the FGDs and KIIs below, we will develop a brief questionnaire encompassing elements of both health systems’ readiness and scalability assessment, which will be conducted with relevant stakeholders prior to their participation in FGDs and KIIs. Ultimately, the questionnaire, FGD, and KIIs will collate key stakeholders’ perceptions of the in-country capacity to translate the knowledge generated from ASCOT into policy and routine practice.

*Normalization Process Theory (NPT)* ^48^

NPT will be used to inform the design of FGDs and KIIs and support both health system readiness and scalability assessment. The NPT and NoMAD tool ^49^ describe four constructs through which stakeholders implement and integrate a new practice into their work: coherence (or sense-making); cognitive participation (or engagement); collective action (work done to enable the intervention to happen); and reflexive monitoring (formal and informal appraisal of the benefits and costs of the intervention) ^48^. Feeding into health systems readiness and scalability assessment, these four constructs will form the backbone of our FGD and KII topic guides (see Extended data ^19^).

*Recruitment of multisectoral stakeholders*

The stakeholder populations are described above. In brief, the subset of people with TB recruited to the pilot trial will be invited to participate in an FGD and workshop at either household visit 2 or 3. As has worked well in Nepal during our previous research, the other purposively selected stakeholder groups will be invited to participate in an FGD or KII, and workshop by phone, email, and/or in person. The discrete consent forms and participant information sheets (PIS) for different participant groups and ASCOT activities can be found in the Extended data ^19^.

*Brief questionnaire*

Prior to participation in FGDs, NTP managers, NTP TB clinic staff, social protection specialists, and ASCOT DPC, CHS and FCHV participants will be asked to complete the brief questionnaire concerning health systems readiness and scalability detailed above. People with TB will not be asked to complete this questionnaire.

*FGDs*

FGDs will be semi-structured and incorporate open-ended questions informed by the work above and topic guides piloted amongst the ASCOT team and in an initial FGD. During the first FGD section, questions will relate primarily to the socioeconomic impact of TB, current (e.g. support for people with DR-TB) and potential socioeconomic support interventions for TB-affected households, and the household level and health systems level challenges to the NTP of delivering them.

The FGDs will be conducted with separate groups of approximately five key stakeholders. Stakeholders will be invited to participate according to their background (e.g. people with TB will be asked to participate in one FGD, and NTP staff will be asked to participate in another separate FGD). The second section of the FGD will include a presentation of the elements that constitute the ASCOT intervention and participants will be asked to discuss their opinions on its potential. Participants who have experienced the intervention, including people with TB diagnosed by ACF or PCF and randomised to an intervention arm, will be asked to discuss their opinions on its successes, challenges, and/or failures, and consider how to refine the intervention to overcome the issues raised. ASCOT DPCs, CHS, and FCHV will be asked to also consider the reasons for non-participation and drop-out amongst the people with TB invited or recruited to participate in their district.

We will perform member checking in each FGD by noting key points of the discussion, summarizing these points on a wall chart, and clarifying their accuracy with the group. Formal field notes may also be taken. Based on our previous work, it is predicted the FGDs will last between 90 and 120 minutes. The FGDs will be moderated by members of the project team trained in qualitative methods including conducting FGDs. The ASCOT field team will support FGDs with people with TB in order to facilitate any dialectic interpretation or contextual explanations. The discussions will be audio recorded in Nepali language, translated into English, and back-translated by an independent translator who is not part of the project team.

*KIIs*

Participants who occupy higher-level positions in terms of policy-making and leadership in either the NTP or social protection programmes in Nepal, will be invited to participate in KIIs. This is in line with our previous research methods, which found attendance and engagement of these participants to be higher for KIIs than FGDs. Given that most of the higher-level agencies, institutions, and leaders are based in Kathmandu, it is likely that this is where the majority of KIIs will take place. Participants will be able to choose the location that suits them best or a location organised by the ASCOT Project Manager. The KIIs will be conducted by the Project Manager and Co-investigator (in Nepali or English) and Chief Investigator (in English) where appropriate. Topic guides for KIIs and subject matter will be parallel to the FGDs described above. 

*Workshop and dissemination meeting*

The final activity will be a one-day workshop bringing together the 40 key stakeholders. The morning section of the workshop will consist of interactive presentations from the project team and stakeholder group representatives, and discussions exploring the preliminary findings of the ASCOT survey, pilot trial, and mixed-methods process evaluation. The afternoon section of the workshop will consist of multi-sectoral working groups (≤10 diverse stakeholders) developing recommendations for refinements of the intervention and considerations ahead of application for funding for the large-scale, well-powered ASCOT trial.

***Randomisation and blinding***

In order to randomise participants into study arms, a random number table will be prepared prior to recruitment by two ASCOT team members (KD, BR). A screenshot will be taken documenting the time and date of the random number table generation in order to improve transparency and reduce the chances of contaminated randomization or tampering. The table will be saved centrally and only accessible by the Project Manager (BR) and no other ASCOT team members.

The random number table will randomise participants 1:1:1:1 to the four study arms (social, economic, socioeconomic, and control arms) in blocks of 32 in each of the four study site districts according to site-level participant identification code. Once participants have given informed consent, been recruited, and given a site-level code, the ASCOT field team member who consented the participant will call the Project Manager (BR) and be informed by either KD or BR to which arm the participant has been allocated.

Digital randomisation using an online programme was originally considered but perceived as inappropriate and unfeasible in the study setting.

It will not be possible to blind participants or ASCOT field team members delivering the interventions (DPC, CHS, FCHV) to the randomisation. However, NTP staff will not be informed of the arm to which participants are randomised.

### Outcomes to be measured

***Co-primary outcomes***

The co-primary outcomes of the ASCOT pilot trial are acceptability and feasibility of the social, economic, and socioeconomic support interventions.

*Feasibility* will be measured using quantitative data including:

i) Recruitment (e.g. number of people invited, recruited, and participant attrition);
ii) Fidelity to survey completion and the intervention (e.g. adherence to and completion of socioeconomic support delivery including receipt of cash transfers and attendance at TB Club amongst intervention arm participants); and
iii) ASCOT staff time and costs plus overall project costs.

*Acceptability* will be measured using mixed-methods including:

i) Quantitative analysis of the implementer and participant satisfaction form;
iii) Thematic analysis of the qualitative FGD and KII data within Sekhon’s Framework for healthcare interventions and quantitative analysis of the brief questionnaire completed prior to FGDs will include evaluation of health systems readiness and scalability ^50, 51^.

***Secondary outcomes***

Unpowered, exploratory secondary outcomes will compare the proportion of people with TB in each study arm by rates of:

- *TB treatment success:* NTP-defined outcome of completed TB treatment or confirmed cured documented in TB register.
- *Catastrophic costs:* TB-affected households that incur total out-of-pocket expenses and lost income during TB illness that equated to more than 20% of the same household’s annual pre-TB income.
- *Wellbeing:* measured by self-reported levels of stigma, depression, and quality of life at six-month follow-up adjusted for baseline.

### Data collection and management

***Survey and pilot trial***

During the survey, information will be collected by the project team during household visits. This information includes but is not limited to socioeconomic, health, psychosocial, and behavioural data. The data of consenting TB patients will subsequently be linked with data from NTP’s TB patient register as part of its routine surveillance data collection in each study site. This data will be collected through use of tablets and/or paper depending on the local situation (e.g. security and feasibility in each district). When entered digitally, results will be entered into a mobile data collection tool via the Commcare – ODK platform (Dimagi, Inc., Boston, MA), which is based on open-source suite of tools called Open Data Kit/OpenRosa developed at the University of Washington's Department of Computer Science and Engineering. The data will be uploaded to secure cloud-based servers hosted by Dimagi. Dimagi servers are secure and HIPAA compliant.

***Process evaluation including FGDs, KIIs, and workshop***

The multisectoral stakeholders identified by the scoping exercise (and also including a subset of purposively sampled people with TB who participated in the survey) will complete a short pre-FGD/KII questionnaire (in person, by post, or by email) and then participate in either an FGD or KII. FGDs and KIIs will be audio-recorded.

All patient data and FGD/KII results will be entered into an encrypted access database on a project computer, which will remain at BNMT secure offices at all times. The data will be uploaded to a LSTM password-secured server.

Following discussions among the ASCOT project team and during TSC meetings, it was decided that a Data Monitoring Committee was not required for this pilot acceptability and feasibility trial with a relatively small sample size. However, a Data Monitoring Committee will be formed for any follow-on large-scale, well-powered trial assessing effectiveness of the intervention on catastrophic costs incurrence and TB treatment outcomes.

Primary study data will be managed by the Data Manager of Birat Nepal Medical Trust. Data input, cleaning, checking and double-checking, and management will be iterative and ongoing throughout the study. Data will be checked for consistency and completeness by the Data Manager and ASCOT Project Manager and double-checked by the Chief Investigator prior to hand-off to LSTM. This data will be exported to Stata and transferred to LSTM servers for analysis. PIS will be provided and written informed consent will be obtained from all study participants. Secondary data obtained from patient register records and other sources of the Nepal NTP will be copied and entered in an electronic database by BNMT staff.

Specifically, audio recordings from the FGDs will be stored in a password-protected digital folder on an LSTM secure server. Only the Chief Investigator, Project Manager, and transcribers will have access to the data. Audio-recording data will be stored for seven years, as per the requirement of the BNMT’s data policy, after which they will be deleted.

Paper-based copies and study documents will be stored in the locked offices of BNMT. The electronic database will be stored in files within LSTM’s secure server and will be password protected. Any tablet devices used for data entry will be password protected and data will be uploaded to LSTM’s secure server weekly. Access to final data will be limited to the Chief Investigator, Data Manager, Project Manager, and key authorised ASCOT staff.

### Data analysis and statistical plan

**Co-primary outcome analyses**

***Feasibility***

Target versus observed participant recruitment, completed cash transfers, completed TB Club attendance, and attrition from follow-up at each survey time point will be reported across study arms using the CONSORT flow diagram, summarised using proportions and percentage rates, and where relevant compared across study arms using a Chi-2 test with p values of <0.05 considered significant.

ASCOT staff and project costs will be summated for the project as a whole and also as total and mean costs by specific project activities (i.e. TB Clubs, home visits, cash transfers). The latter will support generation of estimates of the costs to deliver a single unit activity such as a TB Club or Cash Transfer. Continuous costs data will be summarised by median with interquartile range or mean with standard deviation depending on the distribution of the data. Total ASCOT staff and project costs will be compared across study arms using the Kruskal Wallis test or one way ANOVA with p values of <0.05 considered significant.

***Acceptability***

Quantitative data from implementer and participant feedback forms detail the respondents’ ratings on the provision of ASCOT project activities received including recruitment, TB information and education, home visit, TB club, and cash transfers. Responses are provided in 5-point Likert Scale including 1 “Very bad”, 2 “Bad”, 3 “Neither good nor bad”, 4 “Good”, and 5 “Very Good”. Responses will be summarised using proportions and rates of response by category. Proportions of respondents reporting “Good” and “Very good” in each category will be aggregated and compared across study arms where relevant using a Chi-2 test with p values of <0.05 considered significant.

Qualitative data will be analysed by applying thematic analysis within Sekhon’s framework of healthcare interventions ^51^ and also application to the NoMAD tool’s ^49^ four constructs (coherence, engagement, collective action, and reflexive monitoring) using NVivo 12 to manage the data ^52^. Initial codes will be generated, which will be updated as further data becomes available and collated following each successive FGD and KII respectively. Both open and closed first-order category will be used to label data within NVivo with specific a priori consideration of codes relating to health system readiness and intervention scalability. Codes may then also be grouped together into second-order and third-order themes. After all the transcripts are coded and analysed, to increase trustworthiness, we will independently review coding and themes and refine and triangulate them through further debate and discussion where necessary ^18^.

**Secondary exploratory outcomes analyses**

Key quantitative survey data will include TB-related costs, stigma, mental health, and quality of life. This will be supplemented by TB treatment outcome data from the National TB Program register.

As per our related research, WHO TB Patient Costs Survey methods will be used to calculate total direct costs, lost income, and catastrophic costs (total TB-related costs of more than 20% of the same household’s annual pre-TB income) ^38^. TB treatment outcomes will be collated from National TB Program registers and will be aggregated to TB treatment success (the sum of the National TB Program defined outcomes of “Treatment completion” and “Cure”) vs no TB treatment success (the sum of all other National TB Program defined outcomes including “Lost to follow-up”, “Death”, “Treatment Failure”, “No evaluation”, “Change to drug-resistant TB regimen” and “Transferred out”).

Stigma will be measured by locally developing stigma score for the Nepali context by adapting elements of the validated Van Rie stigma scale, which comprises ten stigma-related questions with 5-point Likert scale responses. Each response category of the scale will be assigned scores of: -2: strongly disagree, -1: disagree; 0: neither agree nor disagree; +1: agree, and +2: strongly agree. The total scores will range from -20 (no stigma) to +20 (highest levels of stigma). A threshold of equal or above the median cohort stigma score will also be used as a binary classification of stigma.

Mental health will be measured using an adapted version of the Patient Health Questionnaires, PHQ-9, which has been validated in Nepal. The tool explores anxiety, depression, wellbeing, and discrimination through nine questions with four-point scale responses of 0: not at all, 1: sometimes, 2: usually, and 3: always. The range of scores is from 0 to 27 and the following categorisations have been used in the validated tool: 1–4 minimal, 5–9 mild, 10–14 moderate, 15–19 moderately severe, and 20–24 severe depression level. A threshold score of equal or above 10 will also be used as a binary classification of depression.

Quality of life will be assessed using the EQ-5D-5L index. EQ-5D-5L uses five dimensions of quality of life: mobility, self-care, usual activities, pain or discomfort, and anxiety or depression. Each question includes a five-category response scale from 1: no problem, 2: slight problem. 3: moderate problem, 4: severe problems, 5: being unable. The EQ-5D-5L has not been validated in Nepal but has been validated in neighbouring India. Since Nepal and India share common sociocultural conditions and geographical features, we will calculated quality of life scores from the Nepal data using the validated value sets and weightings from India as estimates. The EQ-5D-5L also includes a health rating covariate measured on a scale of 0 to 100 based on participants' self-reporting. The participants are asked to choose an appropriate point that best defines their health condition for that day and to label it with a mark on the scale printed in the survey with 0 indicating lowest/poorest health and 100 indicating highest/best health.

Descriptive statistics will be used to analyse the quantitative survey data collected during household visits. In line with our own and others’ related research, the arithmetic means and 95% confidence intervals of patient costs data that is continuous in distribution will be described regardless of the distribution of the data, and any direct expenses, lost income, or annual income recorded as “zero” or missing will be replaced with the mean cost of each costs category (unless >10% of data for a particular variable is missing) ^5, 6, 53^. Nepalese rupees, the local currency, will be converted to United States Dollars according to OANDA rates at the time of data collection. Continuous stigma and quality of life data will be summarised by median and IQR or mean and SD depending on distribution. Categorical data will be summarised as proportions with 95% confidence intervals. Continuous data relating to costs, stigma, mental health, and quality of life scores (including self-rated health) will be compared across study arms where relevant using the Kruskal-Wallis test or one way ANOVA with p values <0.05 considered significant. Proportions of participants above and below the binary thresholds of treatment success, catastrophic costs, stigma, and depression will be summarised and compared across study arms where relevant using a Chi-2 test with p values of <0.05 considered significant.

Statistical analysis will be done using STATA v15 (Statacorp, TX, USA).

### Plans for dissemination of study findings

The intended research outputs of this work are to: i) present the interim and final findings at the International Union Against TB and Lung Disease in November 2022 and 2023 respectively; ii) publish, in 2023, two first-author papers in PubMed citable, peer-reviewed, open-access journals concerning the ASCOT pilot trial; iii) consolidate partnerships with and disseminate findings to key stakeholders (including NTP and TB civil-society), Social Protection Action Research and Knowledge Sharing network (SPARKS, www.sparksnetwork.ki.se) and WHO; and submit a strong application that receives large-scale funding to conduct a well-powered full-scale ASCOT trial that evaluates the impact of the intervention on health and socioeconomic outcomes.

### Trial Steering Committee

In September 2021, a Trial Steering Committee (TSC) was formed to provide oversight and guidance on the ASCOT pilot trial and any future related trials. Potential TSC members were identified by the ASCOT project team according to the National Institution of Health Research “ Good practice guidelines on the recruitment and involvement of public members on Trial Steering Committees (TSCs) / Study Steering Committees (SSCs)”. At the time of selection, the potential TSC members were selected based on their expertise in various fields related to TB, their diverse and complementary skillsets, having no direct research, publications, or outputs with the ASCOT PI in the past five years, and not working in the same department as the ASCOT PI. Email invitations were sent to potential TSC members summarising the ASCOT project, team members, and reiterating key aspects of the Good Practice Guidelines cited above. From responses received, it was possible to create the TSC, which is composed of the following members:

- Dr Ahmad Fuady, Post-doctoral Researcher, Department of Community Medicine, Faculty of Medicine, Universitas Indonesia, Indonesia.
- Professor Buddha Basnyat, Associate Professor, Director of Oxford University Clinical Research Unit-Nepal, hosted by Patan Hospital and the Patan Academy of Health Sciences.
- Dr Bhabhana Shrestha, Tuberculosis Unit, Nepal Anti-Tuberculosis Association/German Nepal TB Project, Kathmandu, Nepal.
- Dr Laura Dean, Post-doctoral Social Science Lecturer, Liverpool School of Tropical Medicine, UK.
- Professor James Lewis, Director of Cardiff University's Y Lab - the Public Services Innovation Lab for Wales, Wales, UK.

### Study status

From August to December 2021, training of BNMT DPC, CHS, and FCHVs took place in the field. In January 2022, recruitment, activities, and the intervention were piloted in 10–20 participants and the initial implementation successes and challenges discussed amongst the project team. Recruitment of consecutively newly diagnosed people with TB in the study sites and key stakeholders continued until September 2022. Write up and dissemination, including a one-day workshop and dissemination meeting of ASCOT team and key stakeholders including the NTP, will be completed prior to the study end date of 31 ^st^ March 2023.

## Discussion

The ASCOT pilot trial extends our Wellcome Trust Seed Award research with diverse stakeholders in Nepal and internationally. This intersectoral participation and broad dissemination through academic meetings, networks such as SPARKS, and partnerships with WHO will continue to ensure the widest possible use of our research findings. Outputs will include publications in leading peer-reviewed scientific journals, media pieces, policy guidance, and a practical handbook on implementing socioeconomic support for TB-affected households. 

The further significance of the study lies in refining a socioeconomic support intervention for TB-affected households in Nepal that is locally-appropriate, feasible, and acceptable. Through collaboration with diverse stakeholders in Nepal from patients to NTP managers to civil-society representatives, it is envisaged that this work will lead onto a successful funding bid for the definitive, well-powered randomised-controlled ASCOT trial, which will evaluate the impact of the intervention on outcome measures including catastrophic costs and TB treatment outcomes.

## Conclusions

Ending TB, alleviating poverty, and eradicating catastrophic healthcare costs are integral aspects of WHO’s global TB policy and the SDGs. This highly timely pilot and future trial will provide the world’s first, robust evidence regarding the feasibility, acceptability, and impact of socioeconomic support for TB-affected households in a TB-endemic LIC. Strong national and international partnerships, collaborations, and networks, will ensure the findings lead to policy and practice change in Nepal and other LICs.

## Data Availability

No underlying data are associated with this article Open Science Framework: Addressing the Social Determinants and Consequences of Tuberculosis in Nepal (ASCOT): a pilot trial. https://doi.org/10.17605/OSF.IO/U5V72 ^19^ This project contains the following extended data: ASCOT CONSENT FORM FOR PARTICIPATION IN SURVEY AND PILOT TRIAL V5.2_27-01-2022_English.pdf ASCOT household contact consentform V5_27-01-2022_English.pdf ASCOT KII and Workshop ConsentForm v4.1 27-Jan-2022_English.pdf ASCOT FGD and workshop consentform-v4.1-27-Jan-2022-English.pdf ASCOT FGD and workshopparticipant information sheet V4.1-27-01-2022.pdf ASCOT KII and workshopparticipant information sheet-V4.1-27-01-2022 English.pdf ASCOT survey and pilot trial participant information sheet-V5-27-01-2022.pdf Household contact pilot trial participant information Sheet v4.1 27 Jan 2022.pdf ASCOT Tuberculosis InformationLeaflet v3-27-01-2022_TW.pdf JGHT ASCOT Survey v5.1 27-01-2022.pdf ASCOT FGD and KII TopicGuides_v4.0 27012022-PR_BR_KD_TW_Clean.pdf ASCOT Protocol v4.3 27012022 TW.pdf ASCOT SOP v11.3 27012022 TW.pdf Nepali_version_Final-v5.231_Jan_2022_Patient cost instrument_(Nepal)_ASCOT project-revised.pdf Data are available under the terms of the Creative Commons Zero "No rights reserved" data waiver (CC0 1.0 Public domain dedication). Open Science Framework: SPIRIT checklist for ‘Protocol for the Addressing the Social Determinants and Consequences of Tuberculosis in Nepal (ASCOT) pilot trial’. https://doi.org/10.17605/OSF.IO/U5V72 ^19^
