# Supplementary material for: Protocol for the Addressing the Social Determinants and Consequences of Tuberculosis in Nepal (ASCOT) pilot trial
Source: Wellcome Open Res. 2022 Dec 13;7:141. Originally published 2022 Apr 26. [Version 3] doi: 10.12688/wellcomeopenres.17669.3 (PMC9975416; doi:10.12688/wellcomeopenres.17669.3)
Supplement: Peer review history

# Peer review: Reviewer response for version 3

**DOI:** 10.21956/wellcomeopenres.20746.r53712

**Published:** 2022-12-13

**Authors:** Louise Marston

**Affiliations:** 1 Research Department of Primary Care and Population Health, University College London, London, UK

Thank you to the authors for modifying the protocol in line with my statistical comments.

Is the study design appropriate for the research question?

Yes

Is the rationale for, and objectives of, the study clearly described?

Yes

Are sufficient details of the methods provided to allow replication by others?

No

Are the datasets clearly presented in a useable and accessible format?

Not applicable

Reviewer Expertise:

Medical statistics

I confirm that I have read this submission and believe that I have an appropriate level of expertise to confirm that it is of an acceptable scientific standard.

# Peer review: Reviewer response for version 2

**DOI:** 10.21956/wellcomeopenres.20625.r53524

**Published:** 2022-12-07

**Authors:** Louise Marston

**Affiliations:** 1 Research Department of Primary Care and Population Health, University College London, London, UK

You are unlikely to have sufficient power to be able to do the multivariable logistic regression analyses you have added to the analysis section. 

 In these analyses you should account for the district the participants were from. It should be possible to do this as a random effect and then use mixed effects logistic regression, but you will still have power problems as in the first paragraph of this report.

Is the study design appropriate for the research question?

Yes

Is the rationale for, and objectives of, the study clearly described?

Yes

Are sufficient details of the methods provided to allow replication by others?

No

Are the datasets clearly presented in a useable and accessible format?

Not applicable

Reviewer Expertise:

Medical statistics

I confirm that I have read this submission and believe that I have an appropriate level of expertise to confirm that it is of an acceptable scientific standard, however I have significant reservations, as outlined above.

# Response

**Published:** 2022-12-07

**Authors:** Tom Wingfield

**Affiliations:** Liverpool School of Tropical Medicine, UK

Dear Dr Marston, Thank you for your further review. We have removed the relevant text concerning regression analyses. Kind regards, ASCOT Team

# Peer review: Reviewer response for version 2

**DOI:** 10.21956/wellcomeopenres.20625.r53525

**Published:** 2022-12-01

**Authors:** Winters Muttamba

**Affiliations:** 1 Makerere University Lung Institute, Makerere University College of Health Sciences, Kampala, Uganda

The authors have attended to my comments. No further comments

Is the study design appropriate for the research question?

Yes

Is the rationale for, and objectives of, the study clearly described?

Yes

Are sufficient details of the methods provided to allow replication by others?

Yes

Are the datasets clearly presented in a useable and accessible format?

Not applicable

Reviewer Expertise:

Tuberculosis research, TB programming

I confirm that I have read this submission and believe that I have an appropriate level of expertise to confirm that it is of an acceptable scientific standard.

# Peer review: Reviewer response for version 1

**DOI:** 10.21956/wellcomeopenres.19547.r52906

**Published:** 2022-10-19

**Authors:** Louise Marston

**Affiliations:** 1 Research Department of Primary Care and Population Health, University College London, London, UK

This is a pilot four arm randomised controlled trial in TB endemic districts of Nepal. Each arm will recruit 32 participants. There is a mixed methods process evaluation embedded into this trial.

 Study design - I am not sure this is an adaptive design (it is not obvious what is being adapted or when). From the description, this sounds more like a pilot randomised controlled trial, with an additional small pilot in each site before the main pilot gets underway. The small initial pilot may or may not result in procedural or other changes to the trial ^1^.

 It is more usual to have one primary outcome and a number of secondary outcomes. I would choose the most important pilot outcome (recruitment or retention) and move the other pilot outcomes to secondary outcomes.

 Unless it is explicitly written in the Participant Information Sheet and the participant consent form, I would advise not to delete quantitative data from the dataset when participants drop out. This is potentially going to make the study underpowered.

 There is little in the statistical analysis section. I am not sure that I could reproduce your intended analyses from the description given.

 Minor point: where you say Data Management Committee, do you mean Data Monitoring Committee?

Is the study design appropriate for the research question?

Yes

Is the rationale for, and objectives of, the study clearly described?

Yes

Are sufficient details of the methods provided to allow replication by others?

No

Are the datasets clearly presented in a useable and accessible format?

Not applicable

Reviewer Expertise:

Medical statistics

I confirm that I have read this submission and believe that I have an appropriate level of expertise to confirm that it is of an acceptable scientific standard, however I have significant reservations, as outlined above.

## References

[1] : Adaptive designs in clinical trials: why use them, and how to run and report them. *BMC Medicine* .2018;16(1) : 10.1186/s12916-018-1017-7 10.1186/s12916-018-1017-7 29490655PMC5830330

# Response

**Published:** 2022-11-15

**Authors:** Tom Wingfield

**Affiliations:** Liverpool School of Tropical Medicine, UK

Dear Dr Muttamba and Dr Marston,

 Thank you both for your constructive reviews of our protocol.

 We have responded to each of your specific comments below and indicate where amendments have been made.

 We are grateful for your comments, which we believe have helped to improve the protocol.

 Yours,

 The ASCOT project team

**---**

 COMMENT 1: Study design - I am not sure this is an adaptive design (it is not obvious what is being adapted or when). From the description, this sounds more like a pilot randomised controlled trial, with an additional small pilot in each site before the main pilot gets underway. The small initial pilot may or may not result in procedural or other changes to the trial1.

 RESPONSE 1: Thank you for the comment and useful reference. We have updated the relevant text in the following sections:

 Study Design: "ASCOT is a randomised-controlled pilot trial and mixed-methods process evaluation of a socioeconomic intervention for people with TB and their households in Nepal. The pilot trial is design to allow adaptation of the intervention in real time during its implementation." 

 Pilot Trial: "ASCOT team members will review the implementation of intervention after this initial pilot and may adapt or refine activities within the intervention. This could include adaptations in dose (e.g. frequency of economic or social support) and mode or mechanism of delivery (e.g. mechanism of economic support could be phone, cash, or bank transfer; social support could be delivered at home, in a community setting, or both)."

 COMMENT 2: It is more usual to have one primary outcome and a number of secondary outcomes. I would choose the most important pilot outcome (recruitment or retention) and move the other pilot outcomes to secondary outcomes.

 RESPONSE 2: Thank you for this comment. In line with our related research and methods used in hybried effectiveness-implementation trials, we have now specified feasibility and acceptability as co-primary outcomes. This option was felt by the ASCOT project team to be preferable to facilitate planned in-country policy dialogue with the National TB Programme and other key stakeholders in Nepal. We have now also specified exploratory, secondary outcomes. The text has been updated as follows:

 Co-primary outcomes:

 "The co-primary outcomes of the ASCOT pilot trial are acceptability and feasibility of the social, economic, and socioeconomic support interventions.

 Feasibility will be measured using quantitative data including:

 • i) Recruitment (e.g. number of people invited, recruited, and participant attrition);

 • ii) Fidelity to survey completion and the intervention (e.g. adherence to and completion of socioeconomic support delivery including receipt of cash transfers and attendance at TB Club amongst intervention arm participants); and

 • iii) ASCOT staff time and costs plus overall project costs.

 Acceptability will be measured using mixed-methods including:

 • i) Quantitative analysis of the implementer and participant satisfaction form;

 • ii) Thematic analysis of the qualitative FGD and KII data within Sekhon’s Framework for healthcare interventions and quantitative analysis of the brief questionnaire completed prior to FGDs will include evaluation of health systems readiness and scalability 50, 51 .

 Secondary outcomes 

 Unpowered, exploratory secondary outcomes will compare the proportion of people with TB in each study arm by

 • TB treatment success: NTP-defined outcome of completed TB treatment or confirmed cured documented in TB register.

 • Catastrophic costs: TB-affected households that incur total out-of-pocket expenses and lost income during TB illness that equated to more than 20% of the same household’s annual pre-TB income.

 • Wellbeing: measured by self-reported levels of stigma, depression, and quality of life at six-month follow-up adjusted for baseline."

 COMMENT 3: Unless it is explicitly written in the Participant Information Sheet and the participant consent form, I would advise not to delete quantitative data from the dataset when participants drop out. This is potentially going to make the study underpowered.

 RESPONSE 3: Thank you for this comment. We have now completed follow-up for the ASCOT pilot trial. Of 132 participants, we had minimal attrition with three participants dropping out during follow-up. This meant that study power remained adequate for both the cost survey (100 participants and above) and feasibility study assessment (the NIHR Research Design Service recommends a sample size of at least 30 to inform sample size parameter estimates with relation to recruitment, retention, and follow-up for larger effectiveness randomised controlled trials. For the subsequent effectiveness ASCOT trial (decision on funding application pending), we will take your comment into account in our data management plan. 

 COMMENT 4: There is little in the statistical analysis section. I am not sure that I could reproduce your intended analyses from the description given.

 RESPONSE 4: Thank you for highlighting this. We have since expanded on the statistical analysis as follows:

 Data Analysis and Statistical Plan:

 "Co-primary outcome analyses 

 Feasibility

 Target versus observed participant recruitment, completed cash transfers, completed TB Club attendance, and attrition from follow-up at each survey time point will be reported across study arms using the CONSORT flow diagram, summarised using proportions and percentage rates, and where relevant compared across study arms using a Chi-2 test with p values of <0.05 considered significant.

 ASCOT staff and project costs will be summated for the project as a whole and also as total and mean costs by specific project activities (i.e. TB Clubs, home visits, cash transfers). The latter will support generation of estimates of the costs to deliver a single unit activity such as a TB Club or Cash Transfer. Continuous costs data will be summarised by median with interquartile range or mean with standard deviation depending on the distribution of the data. Total ASCOT staff and project costs will be compared across study arms using the Kruskal Wallis test or one way ANOVA with p values of <0.05 considered significant.

 Acceptability 

 Quantitative data from implementer and participant feedback forms detail the respondents’ ratings on the provision of ASCOT project activities received including recruitment, TB information and education, home visit, TB club, and cash transfers. Responses are provided in 5-point Likert Scale including 1 “Very bad”, 2 “Bad”, 3 “Neither good nor bad”, 4 “Good”, and 5 “Very Good”. Responses will be summarised using proportions and rates of response by category. Proportions of respondents reporting “Good” and “Very good” in each category will be aggregated and compared across study arms where relevant using a Chi-2 test with p values of <0.05 considered significant.

 Qualitative data will be analysed by applying thematic analysis within Sekhon’s framework of healthcare interventions 51 and also application to the NoMAD tool’s 49 four constructs (coherence, engagement, collective action, and reflexive monitoring) using NVivo 12 to manage the data 53 . Initial codes will be generated, which will be updated as further data becomes available and collated following each successive FGD and KII respectively. Both open and closed first-order category will be used to label data within NVivo with specific a priori consideration of codes relating to health system readiness and intervention scalability. Codes may then also be grouped together into second-order and third-order themes. After all the transcripts are coded and analysed, to increase trustworthiness, we will independently review coding and themes and refine and triangulate them through further debate and discussion where necessary 17 .

 Secondary exploratory outcomes analyses

 Key quantitative survey data will include TB-related costs, stigma, mental health, and quality of life. This will be supplemented by TB treatment outcome data from the National TB Program register.

 As per our related research, WHO TB Patient Costs Survey methods will be used to calculate total direct costs, lost income, and catastrophic costs (total TB-related costs of more than 20% of the same household’s annual pre-TB income) 38 . TB treatment outcomes will be collated from National TB Program registers and will be aggregated to TB treatment success (the sum of the National TB Program defined outcomes of “Treatment completion” and “Cure”) vs no TB treatment success (the sum of all other National TB Program defined outcomes including “Lost to follow-up”, “Death”, “Treatment Failure”, “No evaluation”, “Change to drug-resistant TB regimen” and “Transferred out”).

 Stigma will be measured by locally developing stigma score for the Nepali context by adapting elements of the validated Van Rie stigma scale, which comprises ten stigma-related questions with 5-point Likert scale responses. Each response category of the scale will be assigned scores of: -2: strongly disagree, -1: disagree; 0: neither agree nor disagree; +1: agree, and +2: strongly agree. The total scores will range from -20 (no stigma) to +20 (highest levels of stigma). A threshold of equal or above the median cohort stigma score will also be used as a binary classification of stigma.

 Mental health will be measured using an adapted version of the Patient Health Questionnaires, PHQ-9, which has been validated in Nepal. The tool explores anxiety, depression, wellbeing, and discrimination through nine questions with four-point scale responses of 0: not at all, 1: sometimes, 2: usually, and 3: always. The range of scores is from 0 to 27 and the following categorisations have been used in the validated tool: 1-4 minimal, 5-9 mild, 10-14 moderate, 15-19 moderately severe, and 20-24 severe depression level. A threshold score of equal or above 10 will also be used as a binary classification of depression.

 Quality of life will be assessed using the EQ-5D-5L index. EQ-5D-5L uses five dimensions of quality of life: mobility, self-care, usual activities, pain or discomfort, and anxiety or depression. Each question includes a five-category response scale from 1: no problem, 2: slight problem. 3: moderate problem, 4: severe problems, 5: being unable. The EQ-5D-5L has not been validated in Nepal but has been validated in neighbouring India. Since Nepal and India share common sociocultural conditions and geographical features, we will calculated quality of life scores from the Nepal data using the validated value sets and weightings from India as estimates. The EQ-5D-5L also includes a health rating covariate measured on a scale of 0 to 100 based on participants' self-reporting. The participants are asked to choose an appropriate point that best defines their health condition for that day and to label it with a mark on the scale printed in the survey with 0 indicating lowest/poorest health and 100 indicating highest/best health.

 D

 escriptive statistics will be used to analyse the quantitative survey data collected during household visits. In line with our own and others’ related research, the arithmetic means and 95% confidence intervals of patient costs data that is continuous in distribution will be described regardless of the distribution of the data, and any direct expenses, lost income, or annual income recorded as “zero” or missing will be replaced with the mean cost of each costs category (unless >10% of data for a particular variable is missing) 5, 6, 52 . Nepalese rupees, the local currency, will be converted to United States Dollars according to OANDA rates at the time of data collection. Continuous stigma and quality of life data will be summarised by median and IQR or mean and SD depending on distribution. Categorical data will be summarised as proportions with 95% confidence intervals. Continuous data relating to costs, stigma, mental health, and quality of life scores (including self-rated health) will be compared across study arms where relevant using the Kruskal-Wallis test or one way ANOVA with p values <0.05 considered significant. Proportions of participants above and below the binary thresholds of treatment success, catastrophic costs, stigma, and depression will be summarised and compared across study arms where relevant using a Chi-2 test with p values of <0.05 considered significant.

 Exploratory univariable and multivariable logistic regression models will generate unadjusted and adjusted odds ratios with 95% confidence intervals of the association of socioeconomic and clinical variables, including intervention received, with binary thresholds of treatment success, catastrophic costs, stigma, and depression. Independent variables or subcategories associated with the outcome of interest in the univariable model at a level of p<0·15 will be included in the multivariable model. Interaction terms will be used to evaluate effect modification using the STATA “mfpigen” command and the likelihood ratio test, and how well the final models fit the data will be evaluated using the Akaike Information Criterion.

 Statistical analysis will be done using STATA v15 (Statacorp, TX, USA)."

 COMMENT 5: Where you say Data Management Committee, do you mean Data Monitoring Committee?

 RESPONSE 5: Thank you for highlighting this typographical error, which we have now corrected.

# Peer review: Reviewer response for version 1

**DOI:** 10.21956/wellcomeopenres.19547.r50883

**Published:** 2022-06-14

**Authors:** Winters Muttamba

**Affiliations:** 1 Makerere University Lung Institute, Makerere University College of Health Sciences, Kampala, Uganda

The authors plan to undertake a very important trial. This trial is in line with the End TB strategy and the results have the potential to be used in other low income countries where TB catastrophic costs are still high. 

 A few issues were identified:

1. The eligibility criteria to be made clearer: why are they leaving out TB patients aged under 18 years? Since catastrophic costs are calculated at a household level, the care takers of under 18's do incur costs and it's important to understand to what extent the suggested interventions could protect against these costs. 
2. At what time point will data needed to calculate TB catastrophic costs be collected? Is it at Visit 1, 2 or 3. This is critical to enable extrapolations if patients are in different phases of treatment.
3. The randomisation needs to be expanded. How will randomisation be done, by who?

Is the study design appropriate for the research question?

Yes

Is the rationale for, and objectives of, the study clearly described?

Yes

Are sufficient details of the methods provided to allow replication by others?

Yes

Are the datasets clearly presented in a useable and accessible format?

Not applicable

Reviewer Expertise:

Tuberculosis research, TB programming

I confirm that I have read this submission and believe that I have an appropriate level of expertise to confirm that it is of an acceptable scientific standard.

# Response

**Published:** 2022-11-15

**Authors:** Tom Wingfield

**Affiliations:** Liverpool School of Tropical Medicine, UK

Dear Dr Muttamba and Dr Marston,

 Thank you both for your constructive reviews of our protocol.

 We have responded to each of your specific comments below and indicate where amendments have been made.

 We are grateful for your comments, which we believe have helped to improve the protocol.

 Yours,

 The ASCOT project team

 ---

 COMMENT 1:

 The authors plan to undertake a very important trial. This trial is in line with the End TB strategy and the results have the potential to be used in other low income countries where TB catastrophic costs are still high. 

 RESPONSE 1:

 We are grateful for this positive feedback.

 COMMENT 2:

 The eligibility criteria to be made clearer: why are they leaving out TB patients aged under 18 years? Since catastrophic costs are calculated at a household level, the care takers of under 18's do incur costs and it's important to understand to what extent the suggested interventions could protect against these costs. 

 RESPONSE 2: 

 We agree with the reviewer that it is important to consider the socioeconomic impact on TB-affected households in which the person with TB is under 18 years of age. The study recruitment and follow-up is now complete. We will consider inclusion of children in any larger, follow-on effectiveness trials. 

 COMMENT 3: 

 At what time point will data needed to calculate TB catastrophic costs be collected? Is it at Visit 1, 2 or 3. This is critical to enable extrapolations if patients are in different phases of treatment.

 RESPONSE 3: 

 Thank you for highlighting this important issue. As described in the protocol, data will be collected at three time points of 2-4 weeks, 8-12 weeks, and 20-24 weeks following treatment initiation. This will allow calculations of catastrophic costs over the course of illness to the end of treatment in line with the methods in the World Health Organization TB Patient Cost Survey.

 COMMENT 4:

 The randomisation needs to be expanded. How will randomisation be done, by who?

 RESPONSE 4:

 We have since updated the relevant section of text concerning randomization to read:

 Randomisation and blinding section: 

 "In order to randomise participants into study arms, a random number table will be prepared prior to recruitment by two ASCOT team members (KD, BR). A screenshot will be taken documenting the time and date of the random number table generation in order to improve transparency and reduce the chances of contaminated randomization or tampering. The table will be saved centrally and only accessible by the Project Manager (BR) and no other ASCOT team members.

 The random number table will randomise participants 1:1:1:1 to the four study arms (social, economic, socioeconomic, and control arms) in blocks of 32 in each of the four study site districts according to site-level participant identification code. Once participants have given informed consent, been recruited, and given a site-level code, the ASCOT field team member who consented the participant will call the Project Manager (BR) and be informed by either KD or BR to which arm the participant has been allocated.

 Digital randomisation using an online programme was originally considered but perceived as inappropriate and unfeasible in the study setting.

 It will not be possible to blind participants or ASCOT field team members delivering the interventions (DPC, CHS, FCHV) to the randomisation. However, NTP staff will not be informed of the arm to which participants are randomised."
